# Acceptance and Privacy Perceptions Toward Video-based Active and Assisted Living Technologies: Scoping Review

**DOI:** 10.2196/45297

**Published:** 2023-05-01

**Authors:** Tamara Mujirishvili, Caterina Maidhof, Francisco Florez-Revuelta, Martina Ziefle, Miguel Richart-Martinez, Julio Cabrero-García

**Affiliations:** 1 Department of Nursing University of Alicante Alicante Spain; 2 Communication Science Human-Computer Interaction Center Rheinisch-Westfälische Technische Hochschule Aachen University Aachen Germany; 3 Department of Computer Technology University of Alicante Alicante Spain

**Keywords:** video-based active assisted living technologies, video monitoring, life logging, user acceptance, privacy, older adults, disability, eHealth, virtual assistance, technology, assistive technology, virtual assistant, virtual reality

## Abstract

**Background:**

The aging society posits new socioeconomic challenges to which a potential solution is active and assisted living (AAL) technologies. Visual-based sensing systems are technologically among the most advantageous forms of AAL technologies in providing health and social care; however, they come at the risk of violating rights to privacy. With the immersion of video-based technologies, privacy-preserving smart solutions are being developed; however, the user acceptance research about these developments is not yet being systematized.

**Objective:**

With this scoping review, we aimed to gain an overview of existing studies examining the viewpoints of older adults and/or their caregivers on technology acceptance and privacy perceptions, specifically toward video-based AAL technology.

**Methods:**

A total of 22 studies were identified with a primary focus on user acceptance and privacy attitudes during a literature search of major databases. Methodological quality assessment and thematic analysis of the selected studies were executed and principal findings are summarized. The PRISMA-ScR (Preferred Reporting Items for Systematic Reviews and Meta-Analyses Extension for Scoping Reviews) guidelines were followed at every step of this scoping review.

**Results:**

Acceptance attitudes toward video-based AAL technologies are rather conditional, and are summarized into five main themes seen from the two end-user perspectives: caregiver and care receiver. With privacy being a major barrier to video-based AAL technologies, security and medical safety were identified as the major benefits across the studies.

**Conclusions:**

This review reveals a very low methodological quality of the empirical studies assessing user acceptance of video-based AAL technologies. We propose that more specific and more end user– and real life–targeting research is needed to assess the acceptance of proposed solutions.

## Introduction

As a response to the health care challenges related to an aging society [1] and lack of care personnel [2], technological solutions such as active assisted living (AAL) technologies are being developed and actively funded (eg, [3]), supporting independent living and the quality of life of older adults as well as reducing the need for care personnel and health care costs [4-6]. Based on ambient intelligence, AAL technologies turn living spaces into unobtrusive, flexible, and embedded environments suitable to support older adults with their activities of daily living and to predict risky situations [5]. For these purposes, video-based AAL technology with devices such as red-green-blue (RGB) cameras, thermal cameras, or RGB-depth (RGB-D) devices is particularly appropriate in providing rich sensory information compared to other more traditional sensors such as magnetic sensors or pressure mats [7,8]. RGB cameras are commonly employed color cameras, which capture images using three color channels: red, green, and blue. RGB-D sensors, also known as depth cameras, in addition to capturing color information also measure the distance between the camera and objects in the scene, creating a depth map of the environment. This extra depth information facilitates locating the objects in the scene. Thermal cameras, also known as infrared cameras or thermographic cameras, are a type of camera that detects infrared radiation emitted by objects and converts it into a visible image. Unlike RGB cameras, which capture visible light, thermal cameras can detect temperature differences and produce images based on the heat signatures of objects. Analogous to human sight and owing to sophisticated computer vision methods, these vision-based systems provide ongoing monitoring and are able to analyze the visual data and extract valuable information from them [9].

While AAL is mainly perceived as helpful and beneficial when it comes to assisting older individuals [10-13], the acceptance of the use of cameras in such settings is very limited [14,15] with privacy concerns among the main reasons for such low acceptance [6].

Traditionally, technology acceptance has been measured with the Technology Acceptance Model (TAM) [16] as well as its extension, the Unified Theory of Acceptance and Use of Technology (UTAUT) [17]. Both models are corroborated and widely used, but have been criticized for not enabling context-specific evaluations [18]. Therefore, in the context of AAL technology, other approaches, including frameworks and models, have been proposed that refer to factors influencing the decision of accepting or rejecting AAL technology in specific contexts [10,12,13,19,20]. Peek and colleagues [19] reported the perceived need for technology, social influence, and characteristics of older adults as influencing factors on the acceptance of assistive devices. However, several acceptance studies on AAL suggest that the moment of decision-making is largely determined by trading off potential benefits of using such technologies with potential barriers [6,21,22]. Thereby, prominent benefits are increased safety and security, increased independence, aging in one’s own home, and reduced care burden [19-21,23]. In contrast, important barriers are the lack of human contact, stigmatization, or technical issues [6,20,23]. The barrier with the greatest weight within the tradeoff process is privacy concerns. These concerns arise from the feelings of surveillance, fear of personal data access and misuse, intrusiveness, or the invasion of personal space [6,10,12,21].

With a focus on privacy, Lorenzen-Huber and colleagues [12] developed a framework that includes factors influencing the perception of privacy when adopting home-based ubiquitous technologies. Among these factors included “data granularity,” defined as the level of detail of the data; “data transparency,” which is the extent to which data are visible, verifiable, and controllable; and “data recipient,” indicating with whom data are shared. The authors conclude by mentioning the contextuality, individuality, and older adults’ psychosocial motivation on which privacy concerns depend.

Depending on the unfolding of these relevant factors in this mental tradeoff, positive aspects of technology may even override privacy concerns (eg, [13]). However, when considering duration of use, as shown by Boise et al [24], privacy concerns may increase with the time one uses technology. Moreover, Wilkowska et al [25] found that the relative extent of privacy concerns related to smart assistive technologies depends on the research method used for assessment. The authors reported that, on average, the importance of privacy aspects is highly considered in focus groups, less high in a questionnaires, and tends to be rather unimportant in usability studies.

Overall, the role privacy plays in the acceptance of AAL technologies is complex, can be seen as a tradeoff between barriers and benefits or a multidimensional phenomenon, and its evaluation is dependent on a specific point in time and on the way it is examined. It is therefore timely to review and map the existing literature in this field. Therefore, a scoping review method was applied with the aim of gaining an overview of existing studies examining the viewpoints of older adults (aged≥50 years) and/or their caregivers on technology acceptance and privacy perceptions, specifically toward video-based AAL technology.

The more specific objectives of this review were to (1) scope the body of literature about acceptance and privacy perceptions toward video-based AAL technologies; (2) identify methodologies used to measure acceptance and privacy perceptions toward video-based AAL technology; and (3) identify major knowledge gaps and synthesize the knowledge about perceptions toward video-based AAL technology as a guideline for future research.

This review concentrates on AAL technology that is camera/video-based. The reasons for specifically targeting camera/video are two-fold: on the one hand, visual sensors have high potential to provide quality care [7-9], whereas on the other hand, visual sensors are barely accepted among potential users [14,15]. Ultimately, it is crucial to thoroughly understand these tensions to lower the barriers to acceptance of such effective assistive visual devices and successfully promote actual technology use.

## Methods

### Design

This review was based on a methodological framework developed by Arksey and O’Malley [26] that was subsequently advanced by Peters et al [27], which consists of the following five steps: (1) identifying the research question; (2) identifying relevant studies; (3) study selection; (4) charting the data; and (5) collating, summarizing, and reporting the results. These steps were followed and the PRISMA-ScR (Preferred Reporting Items for Systematic Reviews and Meta-Analyses Extension for Scoping Reviews) guidelines were used as additional guidance [28].

### Literature Search

The final search took place on August 23, 2021 (and a rerun of databases was performed again in September 2022), and was not restricted by publication date. The reproducible full electronic search strategy of all databases searched is provided in Multimedia Appendix 1.

**Figure 1:**
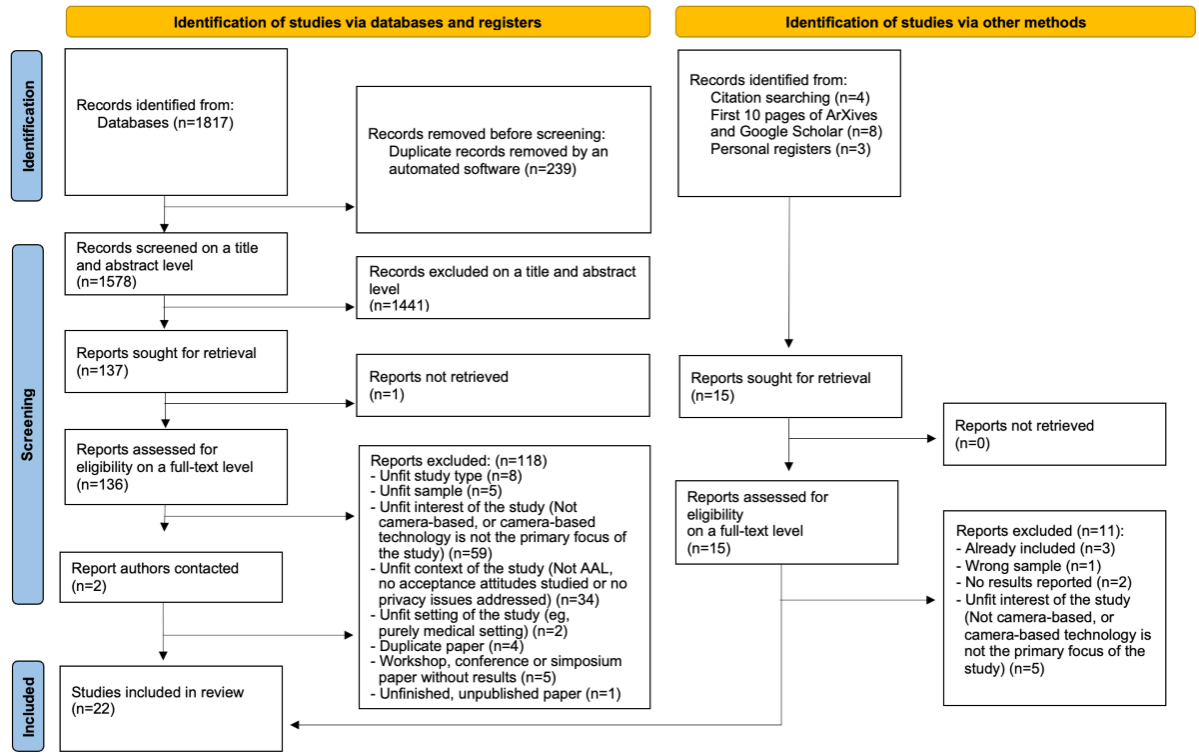
PRISMA (Preferred Reporting Items for Systematic Reviews and Meta-Analyses) 2020 flow diagram of the study selection process [30]. AAL: active and assisted living.

Studies published in English, Spanish, German, French, Portuguese, Italian, Russian, and Georgian languages were considered, although English search terms were used. The following databases were searched: Web of Science (includes Medline), PsycINFO and CINAHL (by EbscoHost), Scopus, Sociology Abstracts by ProQuest, Google Scholar, and ArXives. Articles were included for this review if they dealt with privacy perceptions and acceptance attitudes of potential or current users of video-based AAL technology, such as older adults (50+ years) or disabled people (of any age) and their caregivers, family members, nurses, medical staff, and bystanders (of any age). The full table of inclusion and exclusion criteria can be accessed in Multimedia Appendix 2. A total of 1819 articles were identified through the database search, which were then imported to Rayyan software [29]. Rayyan is a web-based software that allows the import of large numbers of articles, followed by their management and screening for inclusion/exclusion. Using this software, duplicates were removed and two investigators independently screened the articles on a title and abstract level in the first stage. Figure 1 shows the PRISMA (Preferred Reporting Items for Systematic Reviews and Meta-Analyses) diagram of the study selection process [30].

In the next stage, full texts of the 136 articles were retrieved in Mendeley software and were assessed for eligibility on a full-text level by the two researchers independently. Disagreements were resolved by a third researcher and a total of 18 articles were identified for inclusion from the databases searched. Three additional articles were preidentified through personal registers. Reference lists from these publications were manually searched for any reports missed by database searches and personal registers, and one more article was found to be eligible for inclusion in the scoping review, resulting in a total of 22 articles (Table 1).

**Table 1:** Summary of included studies (see Multimedia Appendix 3 for the full table of data extraction).

Study	Country	Design and participants	Technology and setting	Main findings
Bandini et al [31]	Canada	Mixed methods (quantitative online survey and qualitative semistructured interviews); older adults with medical necessity (n=13, age range 46-65 years)	Egocentric wearable camera. Direct contact with technology: participants recorded their daily routine at home using a head-mounted camera over a period of 2 weeks.	61.5%-69.3% of participants expressed little concern about having data of their daily life used by clinicians and researchers for monitoring. Participants would be more comfortable wearing a first-person camera at home than in public. All participants agreed that it was important to start and stop the recordings at any time.
Beach et al [32]	United States	Quantitative (online survey); older adults (1518 disabled and nondisabled adults, age range 45-65 years)	Video systems with/without sound, sensors, motion detectors. No direct contact with technology. Brief description of each technology was presented in the online survey.	Individuals reporting disability had consistently more positive attitudes toward sharing information than those not reporting disability. The level of disability compared to the mere presence of disability influenced the acceptability of technology. Older respondents tended to be more accepting than younger respondents.
Berridge et al. [33]	United States	Mixed methods (online survey with close-ended and open-ended questions); 273 caregivers working in nursing homes or as assisted living providers (no age reported)	Video systems. No direct contact with technology: sample has had diverse exposure to cameras in facilities in the past but no direct contact with technology was deployed in the study.	Most respondents reported the inappropriate invasion of privacy; concerns regarding dignity regarding camera usage; as well as its potential to demoralize, offend, stress, add undue pressure, intimidate, and show lack of confidence in staff. Noted potential advantages were detecting abuse or determining truth in abuse of theft allegations and care quality improvement.
Bourbonnais et al [34]	Canada	Qualitative (exploratory semistructured interviews); 20 care managers, family caregivers, and formal caregivers in five nursing homes (age range 34-70 years)	Intelligent video monitoring system (IVS). No direct contact with technology. A presentation of a potential IVS-integrated app and a short video on the IVS was shown to each participant.	Caregivers thought these tools could improve their well-being at work by improving the behaviors of older people, decreasing the noise in the environment, and reducing the stress and the risk of falls. The risk to confidentiality, cyber dependency, and decreased human contact were also noted by caregivers.
Caine et al [35]	United States	Quantitative (scenario-based online survey); 25 older adults (age range 65-80 years)	Camera, stationary robot (with camera), and a mobile robot (with camera). Direct contact with technology. Participants were given a tour of the tech-aware home and were introduced to the three visual sensing devices (with some privacy-preserving techniques: point-light image, blob image)	The data suggest that privacy concerns are not independent of situation variables. Both device type as well as level of functioning affect privacy concerns in a variety of situations, with privacy concerns being higher when the character in the scenario was high-functioning. Normal video camera images produced more privacy concerns; however, the video camera was rated as more beneficial than the blob tracker.
Caine et al [36]	United States	Mixed methods (quantitative survey, qualitative interviews, and observations); 18 older adults (age range 69-88 years)	Camera, stationary robot (with camera), and a mobile robot (with camera). Direct contact with technology. Participants interacted with the devices in the R-House Living Lab (participants were asked to imagine being in their home).	Older adults in each of the three monitoring device conditions engaged in privacy-enhancing behaviors (PEBs). The camera was the condition in which participants performed the most PEBs. Nine activities were identified, where the comfort with performing household activities decreased with the monitoring devices being present.
Demiris et al [37]	United States	Qualitative (videotaped scenarios followed by in-depth interviews); older adults (10 residents of an independent retirement community, aged >65 years)	Firewire webcam. Direct contact with technology. Participants were filmed while undertaking certain activities at home and recordings after silhouette extractions were shown to them during interviews.	Shape extraction can alleviate privacy concerns associated with the use of cameras. Participants expressed no privacy concerns with silhouette images and emphasized the importance of anonymity in the video sequences. They expressed the desire to control the system by being able to turn it off and on, and also determine who has access to the collected information.
Gelonch et al [38]	Spain	Mixed methods (quantitative self-report questionnaire and qualitative focus groups); older adults with medical necessity and caregivers (N=18, including 9 patients with mild cognitive impairment and medical necessity and their 9 familial caregivers; age range of patients: 65-90 years)	Wearable life-logging camera. Direct contact with technology: participants had to wear the camera (which automatically takes pictures every 30 s) for 7 days throughout the day.	Patients exhibited a good level of acceptance of the camera. However, feelings of embarrassment or worry about the comments that the camera might provoke were reported. Most of the patients and their caregivers reported that they felt relieved when the study ended. Most participants stated that the therapeutic benefits, ease of use, and autonomy of being able to turn the camera off in situations of privacy or discomfort provided sufficient reasons for acceptance.
Gövercin et al [39]	Germany	Qualitative (focus groups); older adults with medical necessity (22 slightly to severely disabled participants with low to severe risk of falling and their caregivers, age range 50-85 years)	Camera systems and motion sensors. No direct contact with technology. Presentation about different information and communication–based technologies (optical and inertial sensors for the prediction and detection of falls at home) during the focus group discussions.	Participants considered a fall prediction system to be as important as a fall detection system. Although the ambient, unobtrusive character of the optical sensor system was appreciated, wearable inertial sensors were preferred because of their wide range of use, which provides higher levels of security. Security and mobility were two major reasons for people at risk of falling to buy the proposed systems.
Harvey et al [40]	United Kingdom	Mixed methods (quantitative questionnaire and qualitative interviews); 6 older adults (mean age 68 years)	Wearable time-lapse camera. Direct contact with technology. Participants wore the equipment for 7 consecutive days during free-living activities.	Participants found the camera to be acceptable to use. They reported that the equipment allowed for sufficient privacy for themselves and others. Regarding reactivity, the equipment had little effect on the participants’ day-to-day lives. Regarding safety, participants felt safe while using the equipment.
Lapierre et al [41]	Canada	Mixed methods (individual interviews that consisted of qualitative and quantitative [questionnaire] assessments); 18 family caregivers (age range 42-87 years)	IVS. No direct contact with technology. Proposed system was explained with a video on the specific technology to each participant before the interview.	Most participants (n=15/18) liked the IVS and were willing to use it. They would worry less if they could be alerted if a care recipient fell, but they were concerned about privacy and cost. Participants had a positive perception of the system and expressed their wishes regarding the kind of alert and the person to contact in case of a fall.
Lapierre et al [42]	Canada	Qualitative (focus groups); 31 professional caregivers, representing home support services for older adults: nurses, social workers, occupational therapists, physiotherapists, physicians, and managers.	IVS. No direct contact with technology: IVS for fall detection and its operation was explained to the participants by showing them videos of 4 different scenarios of fall detection.	Participants reported that the system would provide the caregiver with a quick response to the fall, documentation of its causes, reduction of its consequences and of false emergencies, absence of a device to wear and of an alarm to be given by the caregiver. The system would reassure the carer and give them more freedom.
Lapierre et al [43]	Canada	Qualitative (interviews before and after use of the technology); 6 older women (aged≥65 years)	Programmable video monitoring system. Direct contact with technology: three or four cameras were installed in the bedroom, hallway, and bathroom for 7 consecutive nights and were programmed to record when triggered by movement detection for nighttime slots chosen by the participant. Video images were processed (blurred).	Participants had positive opinions of the video system before the implementation; they appreciated the programmable movement detection during chosen time slots, respecting privacy; the light-emitting diode indicating the recording; and the small cameras. After the experiment, participants reported positive experiences, although some expressed discomfort. Two participants felt uncomfortable receiving visitors during the experiment. Overall, choosing time slots for recording and automatic processing of the images had a positive impact on privacy preservation for participants.
Lapierre et al [44]	Canada	Mixed methods (qualitative interviews and quantitative questionnaires before the implementation, at the midpoint, and at the end); 4 older adults (aged≥65 years) and 4 informal caregivers	IVS. Direct contact with technology: IVS for fall detection was implemented for 2 months at home. In case of a fall, the caregivers received an alert that could include an image of the older adult after the fall. The system had a closed circuit for protecting privacy.	All participants were satisfied with the IVS installation. The caregivers appreciated the fact that the IVS was installed in high-risk zones. However, they did not want the IVS to be installed permanently. Regarding alerts, the older adults liked the image sent to the caregiver so that they could intervene. All caregivers were reassured by receiving images. Finally, all participants appreciated the IVS’s closed-circuit functioning and trusted it to protect their privacy.
Londei et al [45]	Canada	Mixed methods (interviews and quantitative questionnaires); 25 older adults with a history of fall (aged≥65 years)	IVS. No direct contact with technology: 6-minute video including four fall scenarios was presented to the participants that employed IVS for fall detection.	96% of the participants were favorable or partially favorable to the IVS. About half (48%) said that they would use it. The other participants did not wish to use it unless they had been left to live alone or if their health condition worsened. The participants favorable and willing to use the IVS gave two reasons: (1) the sense of confidence and security and (2) the intimacy and privacy given by the system.
Matthews et al [46]	United States	Mixed methods (qualitative interviews and quantitative questionnaires); older adults with medical necessity and their caregivers (9 adults with dementia, age range 73-87 years; 9 family caregivers, age range 44-89 years)	Wearable and wireless camera system. Direct contact with technology: caregiver–care receiver dyads used the system for 3 to 7 days. Caregiver would control when the system was worn and when recording occurred.	Family caregivers gave the technology in general high ratings for making life easy, convenient, and more comfortable, while also reducing privacy and increasing dependency. Their ratings were lower for its role in enabling personal control, safety and security, and interpersonal connectedness, and were the lowest for making life stressful or complicated.
Mulvenna et al [47]	United Kingdom	Mixed methods (semistructured workshops with quantitative questionnaires and group discussions); people with medical necessity and their caregivers (2 people with dementia and 22 caregivers, age range 22-78 years)	Video-camera monitoring system. Direct contact with technology: living lab workshop. A short movie scenario was shown and used as a starting point for a general discussion of the issues raised on the benefits or not of using video surveillance.	Participants supported the concept of the use of a camera in the homes of people living with dementia, with some significant caveats around privacy. The questionnaire reported that 91% found that the idea of a video camera in the home of a person living with dementia living alone was a very good or good idea; 78% considered it very appropriate or appropriate to use cameras in homes of older people generally.
Seelye et al [48]	United States	Mixed methods (qualitative interviews and quantitative questionnaires); 8 older adults and their 8 caregivers from family or friends (age range 64-92 years)	Mobile robot. Direct contact with technology: a mobile, remotely controlled robot with video-communication capability was placed in the home of older adults for 2 complete days.	In general, participants appreciated the potential of this technology to enhance their physical health and well-being, social connectedness, and ability to live independently at home. Participants expressed little concern about privacy, although they highlighted the importance of having control and knowledge of who has access to call them through the device.
Sugihara et al [49]	Japan	Qualitative (interviews); 11 caregivers for people with dementia (age not provided)	Prototype Mimamori cameras. Direct contact with technology: cameras and monitors, with position detection and image capture abilities, were embedded in common spaces except the bathroom and restroom in two group homes.	Positive effects of using the system were: eliminated blind spots in the home and improved working style of caregivers. Negative effects mainly regarded the work stress, as caregivers cannot rest in the break time because of the video recording, and caregivers were heavily stressed about the reduced and violation of privacy rights for themselves, coworkers, and residents.
Wilson et al [50]	United Kingdom	Qualitative (semistructured interviews); 18 older adults (16 with and 2 without chronic pain) and 2 younger participants for comparative analysis between groups (age range 52-81 years)	Wearable camera. Direct contact with technology: a wearable camera was used every day for 7 days. Camera recorded passive images building a visual diary of the day by automatically capturing at least one image every 30 seconds.	Intrusiveness, importance of others, remembering the wearable camera, and ease of use were the main themes that emerged. Initial expectations were that the wearable camera would be intrusive and difficult to use, and that being seen wearing the camera would evoke negative reactions from other people; however, these expectations were contrary to their experiences.
Ziefle et al [51]	Germany	Quantitative (online questionnaires); 165 participants, including 78 males and 87 females (age range 17-94 years)	Video-based system. No direct contact with technology: a medical scenario was presented to participants to introduce them to the field of video-based medical homecare applications.	The results highlight trust and privacy as central requirements, especially when implemented within private spaces. The majority of participants would probably not let medical personnel monitor their home. Most participants would probably accept video-based monitoring systems if they would be helpful. There was a clear answer regarding data protection that must be guaranteed.
Ziefle et al [52]	Germany	Mixed methods (exploratory focus group sessions and a quantitative survey); focus group, n=42 adults (aged 50-73 years) and quantitative survey, n=100 adults (aged 29-93 years)	Microphone, camera, positioning system. No direct contact with the technology. Two short futuristic example movies illustrating integrated ubiquitous technologies were shown in focus groups.	Integration of a camera was not accepted for the bedroom and bathroom in any focus group. Users’ acceptance differed considerably depending on the room type. The main disliked technology type for home monitoring was camera-based systems, followed by the positioning system and the microphone.

### Data Extraction and Critical Appraisal

Data extraction was executed by two researchers independently and was charted in a spreadsheet. The extracted data included author, year, title, place of publication, country, purpose, technology and context of use, methods, and main outcomes. In some cases, the authors of the articles were contacted to obtain and confirm the data. Disagreements and questions during the data extraction process were addressed by the third investigator and interrater agreement was reached through their help. The final table of data extraction is given in Multimedia Appendix 3.

In line with the research objective of understanding the various methods used for assessing acceptance and privacy attitudes toward video-based AAL, the characteristics and methodological quality of the single studies were explored using critical appraisal. An adapted version of the Scale to Assess the Methodological Quality of Studies Assessing Usability of Electronic Health Products and Services [53] was used as a critical appraisal tool for the assessment of acceptance and privacy attitudes of the included studies. All selected articles were examined with 13 binary (yes/no) questions. These questions dealt with aspects such as validity, reliability, coherence, and adequacy of the chosen assessment methods and study designs, with a specific focus on the two constructs of acceptability and privacy. The final version of the critical appraisal tool and its adaptation procedure are provided in Multimedia Appendix 4.

### Data Synthesis and Analysis

The articles were read several times to identify key values and areas in which acceptance and privacy perceptions related to video-based AAL technology appear in the care of people in need. Data were analyzed with thematic analysis [54] using the MAXQDA 2022 qualitative analysis software package [55]. MAXQDA is a software program used for qualitative data analysis that allows researchers to organize, categorize, and analyze large volumes of text, audio, and video data. Guided by the research objectives, a total of 17 codes were identified using this software, which were further grouped into thematic categories. In the labeling process, the terminology used in the studies was respected and no latent meanings were searched. The aim of the synthesis is to present the range of evidence that was identified to meet the objectives of the scoping review.

## Results

### General Characteristics of Included Studies

The 22 included articles were published between 2006 and 2021, with a rather even distribution over the years. Articles were developed from work conducted in 6 countries, with the most publications coming from the United States (n=7, 32%) and Canada (n=7, 32%), followed by Germany (n=3, 14%) and the United Kingdom (n=3, 14%). Most studies were in published in English (n=20, 91%), with the remaining two selected articles published in French.

### Methodology, Design, and Participants

The characteristics of the 22 included studies are summarized in Table 1. Study designs ranged from mixed methods (n=12, 55%) to qualitative (n=7, 32%), as well as quantitative (n=3, 14%) designs. Most of the mixed method studies (10/12, 83%) were composed of a qualitative part such as focus groups or interview assessments and were combined with a quantitative questionnaire that took place at the same time of assessment, except for the studies by Berridge et al [33], Caine et al [35], Lapierre et al [41], and Mulvena et al [47]. The remaining two mixed methods studies combined quantitative and qualitative components within one instrument (ie, interview, online survey). All but one mixed methods study gathered both qualitative as well as quantitative data from the same sample, which resulted in assessments with rather small sample sizes. As for the entirely qualitative studies, the chosen instrument of assessment was either interview (5/7, 71%) or focus group (2/7, 29%). Regarding the purely quantitative studies (n=3, 14%), the instrument of assessment was a questionnaire/survey distributed online in all cases. Acceptance and privacy parameters were studied in three populations: older adults, family caregivers, and health care professionals or professional caregivers. In total, 12 studies evaluated perceptions of technologies of older adults, including 2 studies that included the general population but from the perspective of older adults. Five studies assessed both older adults and their familial caregivers. Health care professionals were examined in four of all selected studies.

### Technology and Settings

More publications based their research on study participants having direct contact with technology (13/22, 59%) than no direct contact with technology (9/22, 41%). Regarding the former, participants either got to experience the relevant assistive technology in a living lab (n=3, 14%) or the technology was installed in a specific environment (n=10, 45%). Where direct contact with technology was not provided, participants were either shown videos (n=5, 23%), introduced to a scenario (n=1, 5%), or the technology was presented to them through a presentation (n=1, 5%).

### Quality Rating

Table 2 shows the results of the quality assessment of the 22 selected studies. The critical appraisal tool used for the assessment was adapted from The Methodological Quality Assessment Guide designed by Silva et al [53] with resulting scores between 1 and 13.

Only 4 of the 22 studies obtained a score of 10 or more out of 13 (maximal score of quality rating) in the critical appraisal assessment. The main questions that failed to fulfill the criteria were related to the triangulation of methods, training and externality of the researcher, as well as the number of participants. The full details of the critical appraisal tool and detailed results are provided in Multimedia Appendix 4.

**Table 2:** Quality assessment of the selected studies (N=22).

Score (out of 13)	Studies, n (%)
10	4 (18)
9	1 (5)
8	6 (27)
7	3 (14)
6	2 (9)
5	1 (5)
4	5 (23)

### Emerging Themes

#### Overview of Themes and Subthemes

Based on previously proposed models of technology acceptance [16,17,19] and the proposed privacy framework [12], the following five categories emerged deductively from thematic analysis, which (with a special focus on privacy) all contribute to the acceptance of video-based AAL: Privacy, Medical Necessity, Social Environment and its influence, Benefits, and Barriers. In detail, the first category (Privacy) is based on the data-related factors of the previously mentioned privacy framework as well as on privacy concerns identified in the literature [6,10,12,21]. The subsequent categories of Necessity and Social Environment and its influence are mostly based on the findings of Peek et al [19]. These are not evaluative categories by nature but are rather dependent on the individual interpretation and can therefore not be attributed to either benefits or barriers. The remaining identified aspects had an evaluative notion and could therefore be classified as either a Benefit or Barrier theme according to common distinctions made in various reviews [6,19,20,23]. Of course, these themes are intertwined and weave within each other; however, the main focus of this scoping review was to address the topics of acceptance and privacy attitudes toward video-based AAL, with privacy having a considerable high impact on acceptance. Therefore, a total of 17 codes identified were distributed into the above-mentioned five categories, which are all expected to be related to and to impact technology acceptance (Textbox 1).

**Thematic categories of the emerged themes in the selected studies.**

Privacy: Informational privacy attitudes and handling and access to the video material

- Intrusiveness
- Type of obtained information
- Location of a video-based active and assisted living system
- Duration of use (control over it)
- Privacy concern mitigation

Necessity: medical necessity

Social environment and its influence

- Negative effects on caregivers
- Bystander
- Positive effect on caregivers and family members

Benefits

- Security and medical safety
- Being independent
- Remain at home

Barriers

- Dignity and confidentiality
- Interference with normal routine
- Cyber dependency
- Decreased human contact

Regarding the overall acceptance of video-based AAL, the results from the selected 22 studies show that acceptance attitudes toward video-based AAL technologies are rather conditional. Few studies reported more concerns over the use of monitoring systems than advantages [33,49]; however, most of the studies note that depending on diverse variables, the acceptance of video-based AAL technologies also vary. Generally, positive attitudes toward technology were related to greater acceptance of sharing and recording health-related information. Another general tendency across studies observed was that even when participants rated the proposed system as favorable, this would not always translate into their willingness to use it. They thought they would rather adopt it in the future if they were living alone, were more impaired, were at greater risk of falling, or that they were independent and did not yet need the proposed technology.

#### Privacy

##### Informational Privacy Attitudes and Handling and Access to the Video Material

Most of the studies reported much higher levels of trust in health care providers than in insurance companies and the government. Selected studies also show that participants agree that relatives, family members, and health care providers can have access to the video material, although there is slight interstudy variation in this regard. For example, Bourbonnais et al [34] demonstrated that participants wish for a user code of ethics to be developed and that older people or their families should be asked to provide consent for the use of the technologies, and suggested including a feature that would make the technologies inaccessible to staff outside the health care center. Respondents across the studies expressed fear of third parties accessing the visual information, threats to data security, and susceptibility to hacking. Likewise, the importance of having control and knowledge of who has access to what information was highlighted across the studies. Interestingly, Lapierre et al [43] demonstrated a preference for each camera having a storage card rather than using the cloud as a data repository. According to Beach et al [32], positive attitudes toward technology in general was the strongest predictor in all of the models tested, accounting for between 2.0% and 4.9% of the unique variance in informational privacy attitudes.

##### Intrusiveness

Invading physical as well as emotional privacy was reported as worrisome across the studies by the participants. Ziefle et al [52] reported that negative aspects of being watched were frequently compared to future visions of Orwell’s “1984, Big Brother is watching you.” Among the selected studies, only Bandini et al [31] investigated participants’ perceptions prior to and after the use of a camera system. Interestingly, expectations and worries about the intrusiveness of the camera being an observational tool were not borne out after having used the device. Wilson et al [50] reported that participants having control of the images produced by the system may have reduced participants’ anxiety over the intrusiveness of the images themselves.

##### Type of Obtained Information

The privacy-by-design paradigm in technology allows data protection through inherent technology design [56]. Six of the 22 selected studies used different kinds of privacy-by-design techniques for privacy preservation [34,35,37,43-45]. These techniques allow automatic processing of images so that the obtained visual information is filtered to guarantee privacy, such as customized blurred or silhouette images. In addition to these privacy filters, some of the studies also offered a closed‐circuit system of the monitoring systems, where the videos were not publicly accessible and only transmitted after a relevant or emergency event. Some privacy filters were more acceptable than others; for example, participants of the study by Demiris et al [37] saw more value in 2D silhouettes versus 3D human representations. However, it is important to note that automatic processing of the visual information as well as closed-circuit cameras had a positive impact on the acceptance of video-based AAL technologies in all the studies listed above, as participants trusted it to protect their privacy.

##### Location of a Video-based AAL System

Most studies showed that bathrooms and bedrooms are the areas where the installation of video-based AAL systems is accepted the least by individuals. Demiris and colleagues [37] suggested that preferences for the location of a monitoring system may depend on gender or other characteristics and participants would prefer to have control over choosing the location of the device installation. Interestingly, Gövercin et al [39] reported that participants with a fall history or experience of falls (relative or neighbor) stated that they would even accept the installation of optical sensors in the bathroom or bedroom. In line with this, 8 of the 19 participants of the study by Londei and colleagues [45] would have accepted being videotaped in the bedroom and bathroom if the obtained images were modified with privacy filters.

##### Duration of Use (Control Over Technology)

Most of the selected studies conveyed the idea that it is very important for individuals to have control over the duration of use of a video-based AAL system; in particular, they wish to turn it on and off whenever they like. Participants believed that this ability of a system was an advantage that also maintains privacy. Likewise, most of the studies showed that participants were not happy with the idea of a permanent installation of a monitoring system without them having control over the duration of its use.

##### Privacy Concern Mitigation

Participants across the studies identified several positive aspects that helped prevent privacy concerns, including privacy filters that guaranteed anonymity of the filmed subjects; having control over the system in terms of choosing its location and time for recording; and in the case of wearable cameras, the egocentric point of view of the camera, which did not show the user’s face alleviated participants’ worries. Moreover, studies showed that the greater the perceived need for help, the more privacy one may be willing to give up.

#### Necessity

Medical necessity was reported as one of the greatest predictors and modulators of acceptance attitudes toward video-based AAL technologies across all included studies. The perceived benefit-to-cost ratio appeared to increase with the level of medical necessity, which also included more readiness to share information or accept potential privacy threats.

#### Social Environment and Its Influence

##### Negative Effects on Caregivers

Some studies mainly identified possible negative effects on formal caregivers, such as feeling threatened by being under constant surveillance [33,36,49]. These studies also highlighted the violation of the privacy rights of caregivers. Berridge et al [33] further noted that some participants had reported a potential of the monitoring system to contribute to a culture of mistrust, along with its potential to demoralize, offend, stress, add undue pressure, intimidate, and show a lack of confidence in staff that could ultimately impede care relationships.

##### Bystander

Most participants across the studies expressed concerns about the presence of household members, visitors, roommates, or facility staff in the video. Some of them felt uncomfortable receiving visitors, coupled with the fear of explaining the installed system to them. The need for consent from the bystanders was raised. Interestingly, two studies using a wearable camera [40,50] reported that participants had very little reaction and comments from others with regard to wearing the camera in contrast to their expectation. Participants across the studies also addressed a need for control over the time and location of such monitoring to avoid violating bystander privacy.

##### Positive Effect on Caregivers and Family Members

Half of the selected studies integrated caregivers, including formal caregivers from institutions or family members. The thematic analysis demonstrated that a video-based AAL system could alleviate anxiety in caregivers by detecting medical emergencies, could give them more peace of mind, and increase their well-being in general. Caregivers from the facilities also noted that their workload could be greatly alleviated using these technologies, which would also result in better care practices. However, caregivers also noted the possible negative effects of video-based AAL technologies, which are described in further detail in the Barriers section below.

#### Benefits

##### Security and Medical Safety

All selected studies agreed that the biggest benefit of video-based AAL technology was detecting medical emergencies, which then leads to feelings of security and peace of mind. People are willing to accept surveillance, and even the loss of privacy it entails, when the result is greater security of person and property and/or faster response to emergency situations. Apart from detecting emergencies, some studies also reported a great benefit in the documentation of emergencies or explanation of falls, such as checking where the resident is hit in the falling accident [37,49]. Importantly, Berridge and colleagues [33] noted that the most commonly raised potential advantage by the participants was the use of cameras as a tool to detect abuse or determine truth in abuse or theft allegations, which would also mean correcting staff behaviors.

##### Being Independent

Participants across the studies agreed that proposed video-based AAL technologies promoted more autonomy and the ability to live more independently whether in their own home or a residential facility. In the case of living independently in their own homes, proposed technologies reduced their fear of being alone at home and promoted a sense of confidence and security.

##### Remain at Home

The ability to be autonomous and independent led participants to appreciate the possibility to remain in their own homes and feel safe.

#### Barriers

##### Overview

With privacy being the main threat in the video-based AAL technologies, we here present some other barriers identified through the thematic analysis. Besides the two main barriers presented below, two other less prominent barriers identified were the potential of cyber dependency and decreased human contact.

##### Dignity and Confidentiality

The risk to confidentiality and concern over dignity was often raised across the studies in relation to the video-based AAL technology. Feelings of vulnerability, embarrassment, “feeling stupid,” or the potential of monitoring technologies to demoralize them and arouse negative emotions were also reported among participants of the selected studies.

##### Interference With Normal Routine

Participants changing their behaviors because of the technology in question was detected across the studies. Caine et al [36] executed an extensive study on privacy-enhancing behaviors, and reflected that the 4 activities with the greatest decrease in comfort level while being monitored were related to being nude with 3 of the 4 activities related to intimate activities, resulting in the conclusion that privacy concerns lead older adults to change behavior in a home environment when they are monitored by a variety of monitoring devices.

## Discussion

Video-based AAL technology acceptance research still has a long way to go. This scoping review managed to grasp some important points of this process. We synthesized existing knowledge about the benefit-barrier tradeoff of video-based AAL technologies, and further identified knowledge gaps and directions for future research.

To synthesize existing knowledge on attitudes and perceptions of technology acceptance and privacy, a thematic analysis across the 22 selected studies was performed. Five main categories emerged in this process: Privacy, Medical Necessity, Social Environment and its influence, as well as separately grouped Benefits and Barriers. The latter two themes are usually weighted off against each other and hence play a crucial role when it comes to the decision of accepting or rejecting assistive technology [21]. While most of the Benefit and Barrier subcategories are in line with those mentioned in the preimplementation acceptance model [19], the thematic analysis of this study revealed a new subcategory, named *interference with the normal routine*. This subcategory, identified as a barrier, might be specific for video-based AAL. In fact, behavior changes and discomfort due to video surveillance are reported. Indeed, a camera, analogous to the human eye, can “see” and report data that are easily interpretable by humans compared to numerical data. Therefore, the fear of the risk to perhaps accidentally reveal personal and intimate details is particularly high.

Privacy, as the main emerging category, presented not only as a notion of threat or concern, but further branched into six identified subcategories depicting multiple aspects of the construct and demonstrating that privacy attitudes are rather conditional and tradeable. Similarly, in the model of preimplementation acceptance [19], privacy is part of the concerns influencing technology acceptance. However, the authors also note the willingness of users to trade privacy for major benefits of technology use. While privacy is not specifically mentioned in models such as the TAM or UTAUT [16,17], the privacy framework proposed by Lorenzen-Huber et al [12] bears parallels with several privacy subcategories emerging in this study. For instance, the dimension of “data granularity” emphasizes the contextuality and importance of data transparency when it comes to the acceptable amount of data being collected, compiled, and communicated [12]. In addition, the subcategory *informational privacy attitudes and handling and access to the video material* that emerged in our review highlighted the importance of controlling the data as well as the data flow. Data access is viewed as critical depending on the person with access to the data (ie, family member or external). Efforts to preserve (data) privacy are being developed as part of the so-called privacy-by-design paradigm, as described under the subcategory *type of obtained information*. The subcategory *location of video-based AAL system* states that typically sensitive activities occurring in the bedroom and bathroom particularly evoke privacy concerns. Lorenzen-Huber et al [12] reported a general trend in their dimension called “sensitivity of activity” that any given space bears different privacy needs depending on the activity performed.

Previous models [17,19,57] have identified social influence as “the degree to which an individual perceives that important others believe he or she should use the new system.” The category of *social environment and its influence* emerging in this study is not entirely based on this notion, but highlights the dynamics within the social environment that can have an influence on acceptance. There are two potential identified subcategories that illustrate these dynamics. First, *negative effects on caregivers* unfolds the fear that video-based AAL technology may contribute to a culture of mistrust, whereas *positive effect on caregivers and family members* explains how video-based AAL may alleviate anxiety in caregivers and provide more peace of mind. Another emerging topic, *concerns regarding bystanders*, seems to considerably influence participants’ perceptions of video-based AAL. This concern might be particularly relevant when it comes to camera technology due to pervasive monitoring and the visibility of the system.

With so many factors playing a role in the acceptance of video-based AAL systems, specific questions based on real-life practice need to be addressed in future studies to obtain an accurate picture of user acceptance. During our literature search, tens of general technology acceptance studies were identified; however, only 22 of them focused specifically on video-based AAL acceptance. This is important to note, as each technological solution has its own specifications and it is very difficult to talk about general technology acceptance. This is particularly evident since even when considering only video-based AAL technologies, acceptance depends on numerous variables. Hence, more specific, end user–focused, and real life–targeting research needs to be done, where the type of technology, obtained data thresholds, control over the system, and user specificities such as context and needs are taken into account. This is particularly evident given that in 9 of the selected 22 studies, the participants did not even have any direct contact with the proposed technology when answering questions about its acceptance. This point can be taken to another level by observing an interesting tendency across the studies: even when participants rated the proposed system as favorable, this would not always translate into their willingness to use it. This discrepancy between attitudes and behaviors has been well-documented for a long time [58] and the same seems to hold in terms of privacy attitudes and privacy-related behavior, a notion known as the privacy paradox [59]. Out of the 22 studies included in this review, only one examined participants’ attitudes before and after the use of technology [43]. We propose that more pre-post studies are needed to obtain a clear picture of this matter. These are not the only methodological weaknesses found in the selected publications. Even if the majority of the studies combined qualitative and quantitative methods to draw their conclusions (54.5%), a considerable number of publications used only one method in their study (qualitative only, 31.8%; quantitative only, 13.6%). This review also manifests that existing empirical studies on technology acceptance lack a unified theoretical framework, such that only four of the selected studies [38,43,50,52] refer to the main existing models of technology acceptance [16,17]. Interestingly, despite the fact that the included articles were developed from work conducted in 6 countries, no cross-cultural tendencies were identifiable based on the selected studies. Hence, there is a need for more cross-cultural studies.

Overall, it is very important to note that the panorama of published studies reveals a tremendous methodological weakness, as they fail to consider and report relevant methodological aspects. Research in this area still seems to be in an exploratory stage; hence, more effort is needed to take off from this phase. Taking all this information into account, studies of higher methodological quality targeted at specific technologies are needed to answer the questions of user acceptance and privacy in video-based AAL.

## Appendix

Multimedia Appendix 1 Search strategy.

Multimedia Appendix 2 Inclusion and exclusion criteria.

Multimedia Appendix 3 Data extraction.

Multimedia Appendix 4 Critical appraisal tool and adaptation procedure.

## Abbreviations

AAL: active and assisted living
PRISMA: Preferred Reporting Items of Systematic Reviews and Meta-Analyses
PRISMA-ScR: Preferred Reporting Items of Systematic Reviews and Meta-Analyses Extension for Scoping Reviews
RGB: red, green, blue
RGB-D: red, green, blue with depth
TAM: Technology Acceptance Model
UTAUT: Unified Theory of Acceptance and Use of Technology
