# Supplementary material for: Acceptance and Privacy Perceptions Toward Video-based Active and Assisted Living Technologies: Scoping Review
Source: J Med Internet Res. 2023 May 1;25:e45297. doi: 10.2196/45297 (PMC10186188; doi:10.2196/45297)
Supplement: Multimedia Appendix 1 [file jmir_v25i1e45297_app1.docx]

**Appendix 1 - Search Strategy**

| **P** | **I** | **Co** |
| --- | --- | --- |
| **Population or Problem** | **Interest** | **Context** |
| What are the characteristics of the Population or the patient?  What is the **P**roblem, condition or disease you are interested in? | **I**nterest relates to a defined event, activity, experience or process | **Co**ntext is the setting or distinct characteristics |

*Source:* [*https://libguides.murdoch.edu.au/systematic/PICO*](https://libguides.murdoch.edu.au/systematic/PICO)

***Perceptions of personal safety and privacy in frail elderly, disabled people and their caregivers in the context of video-based lifelogging technologies*.**

P **Frail elderly, disabled people, caregivers**

*Related: elderly, aged, older adults, aging, disabled, chronically ill, caregivers, in need of care, care professionals, medical staff, care experience*

I **Perception of Personal safety and Privacy**

*Related: perception, attitude, preference, acceptance, technology acceptance, privacy, visual privacy protection, personal information, information privacy, privacy behaviour, privacy paradox, privacy calculus, willingness to disclose*

CO **Video-based** **Lifelogging technologies**

*Related: video, camera, surveillance, surveillance camera, video surveillance, video monitoring, monitoring, monitoring technology, camera-based technologies, video-based technologies, lifelogging, lifelogging cameras,* *visual technologies*

**Keywords:**

ambient assisted living, AAL, active assisted living, active and healthy aging, assistive needs, aging, aged, elderly, aging in place, independent living, maintaining independence, lifelogging, lifelogging technologies, assistive technologies, computer vision, image processing, gerontechnology, intelligent systems, video surveillance, medical monitoring, health monitoring, video cameras, in-home monitoring, smart homes, medical safety, e-health, telecare, acceptance, perception, technology acceptance, perceived benefits and barriers, perceived sensitivity, personal information, privacy, information privacy, privacy protection, visual privacy protection, privacy behaviour, data security, anonymization, privacy paradox

**Databases searched:**

- **Web of Science (includes Medline)**
- **PsycINFO and CINAHL (by EbscoHost)**
- **Scopus**
- **Sociology Abstracts by ProQuest**
- **Google Scholar**
- **ArXives**

**Web of Science (includes Medline)**

***1,492 results from Web of Science Core Collection, MEDLINE® :***

*N.B. Exact search was executed*

**#1**

TI=(elderly OR aged OR older adult* OR disabled OR chronic* ill* OR caregiver* OR in need of care OR care professional* OR medical staff ) OR AB=(elderly OR aged OR older adult* OR disabled OR chronic* ill* OR caregiver* OR in need of care OR care professional* OR medical staff )

**#2**

TI=(perception* OR attitude* OR technology acceptance OR privacy OR visual privacy protection OR personal information OR information privacy OR privacy behavio$r OR privacy paradox OR privacy calculus OR willingness to disclose) OR AB= (perception* OR attitude* OR technology acceptance OR privacy OR visual privacy protection OR personal information OR information privacy OR privacy behavio$r OR privacy paradox OR privacy calculus OR willingness to disclose)

**#3**
TI=(video* OR camera* OR surveillance camera* OR video surveillance OR video$monitoring OR monitoring technolog* OR camera-based technolog* OR video-based technolog* OR lifelogging camera*) OR AB=(video* OR camera* OR surveillance camera* OR video surveillance OR video$monitoring OR monitoring technolog* OR camera-based technolog* OR video-based technolog* OR lifelogging camera*)

**#4**

TS=( ambient assisted living OR AAL OR active assisted living OR active and healthy aging OR assistive needs OR aging in place OR independent living OR maintaining independence OR lifelogging OR lifelogging technolog* OR assistive technolog* OR computer vision OR image processing OR gerontechnology OR video surveillance OR medical monitoring OR health monitoring OR video camera* OR in-home monitoring OR smart home* OR e$health OR telecare OR technology acceptance OR perceived benefit* and barrier* OR perceived sensitivity OR personal information OR privacy OR information privacy OR privacy protection OR visual privacy protection OR privacy behaviour OR data security OR anonymization OR privacy paradox )

**(((#1) AND #2) AND #3) AND #4**

**PsycINFO and CINAHL (by EbscoHost)**

***56 results***

AB ( ( elderly OR aged OR “older adult*” OR disabled OR “chronic* ill*” OR caregiver* OR “in need of care” OR “care professional*” OR “medical staff” ) ) AND AB ( ( perception* OR attitude* OR “technology acceptance” OR privacy OR “visual privacy protection” OR “personal information” OR “information privacy” OR “privacy behavio?r” OR “privacy paradox” OR “privacy calculus” OR “willingness to disclose” ) ) AND AB ( ( video* OR camera* OR “surveillance camera*” OR “video surveillance” OR “video?monitoring” OR “monitoring technolog*” OR “camera-based technolog*” OR “video-based technolog*” OR “lifelogging camera*”) ) AND SU ( ( “ambient assisted living” OR AAL OR “active assisted living” OR “active and healthy aging” OR “assistive needs” OR “aging in place” OR “independent living” OR “maintaining independence” OR lifelogging OR “lifelogging technolog*” OR “assistive technolog*” OR “computer vision” OR “image processing” OR gerontechnology OR “video surveillance” OR “medical monitoring” OR “health monitoring” OR “video camera*” OR “in-home monitoring” OR “smart home*” OR e?health OR telecare OR “technology acceptance” OR “perceived benefit* and barrier*” OR “perceived sensitivity” OR “personal information” OR privacy OR “information privacy” OR “privacy protection” OR “visual privacy protection” OR “privacy behaviour” OR “data security” OR anonymization OR “privacy paradox” ) )

***2 results***

TI ( ( elderly OR aged OR “older adult*” OR disabled OR “chronic* ill*” OR caregiver* OR “in need of care” OR “care professional*” OR “medical staff” ) ) AND TI ( ( perception* OR attitude* OR “technology acceptance” OR privacy OR “visual privacy protection” OR “personal information” OR “information privacy” OR “privacy behavio?r” OR “privacy paradox” OR “privacy calculus” OR “willingness to disclose” ) ) AND TI ( ( video* OR camera* OR “surveillance camera*” OR “video surveillance” OR “video?monitoring” OR “monitoring technolog*” OR “camera-based technolog*” OR “video-based technolog*” OR “lifelogging camera*”) ) AND SU ( ( “ambient assisted living” OR AAL OR “active assisted living” OR “active and healthy aging” OR “assistive needs” OR “aging in place” OR “independent living” OR “maintaining independence” OR lifelogging OR “lifelogging technolog*” OR “assistive technolog*” OR “computer vision” OR “image processing” OR gerontechnology OR “video surveillance” OR “medical monitoring” OR “health monitoring” OR “video camera*” OR “in-home monitoring” OR “smart home*” OR e?health OR telecare OR “technology acceptance” OR “perceived benefit* and barrier*” OR “perceived sensitivity” OR “personal information” OR privacy OR “information privacy” OR “privacy protection” OR “visual privacy protection” OR “privacy behaviour” OR “data security” OR anonymization OR “privacy paradox” ) )

**Scopus**

***261 results***

( TITLE-ABS ( elderly OR aged OR “older adult*” OR disabled OR “chronic* ill*” OR caregiver* OR “in need of care” OR “care professional*” OR “medical staff” ) )

AND ( TITLE-ABS ( perception* OR attitude* OR “technology acceptance” OR privacy OR “visual privacy protection” OR “personal information” OR “information privacy” OR “privacy behavio?r” OR “privacy paradox” OR “privacy calculus” OR “willingness to disclose” ) )

AND ( TITLE-ABS ( video* OR camera* OR “surveillance camera*” OR “video surveillance” OR “video?monitoring” OR “monitoring technolog*” OR “camera-based technolog*” OR “video-based technolog*” OR “lifelogging camera*”) )

AND ( KEY ( “ambient assisted living” OR AAL OR “active assisted living” OR “active and healthy aging” OR “assistive needs” OR “aging in place” OR “independent living” OR “maintaining independence” OR lifelogging OR “lifelogging technolog*” OR “assistive technolog*” OR “computer vision” OR “image processing” OR gerontechnology OR “video surveillance” OR “medical monitoring” OR “health monitoring” OR “video camera*” OR “in-home monitoring” OR “smart home*” OR e?health OR telecare OR “technology acceptance” OR “perceived benefit* and barrier*” OR “perceived sensitivity” OR “personal information” OR privacy OR “information privacy” OR “privacy protection” OR “visual privacy protection” OR “privacy behaviour” OR “data security” OR anonymization OR “privacy paradox” ) )

**Sociology Abstracts by ProQuest**

***5 results***

ab(( elderly OR aged OR “older adult*” OR disabled OR “chronic* ill*” OR caregiver* OR “in need of care” OR “care professional*” OR “medical staff” ) ) AND ab(( perception* OR attitude* OR “technology acceptance” OR privacy OR “visual privacy protection” OR “personal information” OR “information privacy” OR “privacy behavio?r” OR “privacy paradox” OR “privacy calculus” OR “willingness to disclose” )) AND ab(( video* OR camera* OR “surveillance camera*” OR “video surveillance” OR “video?monitoring” OR “monitoring technolog*” OR “camera-based technolog*” OR “video-based technolog*” OR “lifelogging camera*”)) AND su(( “ambient assisted living” OR AAL OR “active assisted living” OR “active and healthy aging” OR “assistive needs” OR “aging in place” OR “independent living” OR “maintaining independence” OR lifelogging OR “lifelogging technolog*” OR “assistive technolog*” OR “computer vision” OR “image processing” OR gerontechnology OR “video surveillance” OR “medical monitoring” OR “health monitoring” OR “video camera*” OR “in-home monitoring” OR “smart home*” OR e?health OR telecare OR “technology acceptance” OR “perceived benefit* and barrier*” OR “perceived sensitivity” OR “personal information” OR privacy OR “information privacy” OR “privacy protection” OR “visual privacy protection” OR “privacy behaviour” OR “data security” OR anonymization OR “privacy paradox” ))

***1 result***

ti(( elderly OR aged OR “older adult*” OR disabled OR “chronic* ill*” OR caregiver* OR “in need of care” OR “care professional*” OR “medical staff” ) ) AND ti(( perception* OR attitude* OR “technology acceptance” OR privacy OR “visual privacy protection” OR “personal information” OR “information privacy” OR “privacy behavio?r” OR “privacy paradox” OR “privacy calculus” OR “willingness to disclose” )) AND ti(( video* OR camera* OR “surveillance camera*” OR “video surveillance” OR “video?monitoring” OR “monitoring technolog*” OR “camera-based technolog*” OR “video-based technolog*” OR “lifelogging camera*”)) AND su(( “ambient assisted living” OR AAL OR “active assisted living” OR “active and healthy aging” OR “assistive needs” OR “aging in place” OR “independent living” OR “maintaining independence” OR lifelogging OR “lifelogging technolog*” OR “assistive technolog*” OR “computer vision” OR “image processing” OR gerontechnology OR “video surveillance” OR “medical monitoring” OR “health monitoring” OR “video camera*” OR “in-home monitoring” OR “smart home*” OR e?health OR telecare OR “technology acceptance” OR “perceived benefit* and barrier*” OR “perceived sensitivity” OR “personal information” OR privacy OR “information privacy” OR “privacy protection” OR “visual privacy protection” OR “privacy behaviour” OR “data security” OR anonymization OR “privacy paradox” ))

**Arxiv (computer science)**

***10 X 7 Results***

Abstract

elderly OR aged OR “older adult*” OR disabled OR “chronic* ill*” OR caregiver* OR “in need of care” OR “care professional*” OR “medical staff”

Abstract

perception* OR attitude* OR “technology acceptance” OR privacy OR “visual privacy protection” OR “personal information” OR “information privacy” OR “privacy behavio$r” OR “privacy paradox” OR “privacy calculus” OR “willingness to disclose”

Abstract

video* OR camera* OR “surveillance camera*” OR “video surveillance” OR “video?monitoring” OR “monitoring technolog*” OR “camera-based technolog*” OR “video-based technolog*” OR “lifelogging camera*”

ALL Fields

“ambient assisted living” OR AAL OR “active assisted living” OR “active and healthy aging” OR “assistive needs” OR “aging in place” OR “independent living” OR “maintaining independence” OR lifelogging OR “lifelogging technolog*” OR “assistive technolog*” OR “computer vision” OR “image processing” OR gerontechnology OR “video surveillance” OR “medical monitoring” OR “health monitoring” OR “video camera*” OR “in-home monitoring” OR “smart home*” OR e$health OR telecare OR “technology acceptance” OR “perceived benefit* and barrier*” OR “perceived sensitivity” OR “personal information” OR privacy OR “information privacy” OR “privacy protection” OR “visual privacy protection” OR “privacy behaviour” OR “data security” OR anonymization OR “privacy paradox”

*N.B. First ten pages were screened*

**PsyArxiv**

***10 X 8 Results***

(elderly OR aged OR “older adult*” OR disabled OR “chronic* ill*” OR caregiver* OR “in need of care” OR “care professional*” OR “medical staff” ) AND (perception* OR attitude* OR “technology acceptance” OR privacy OR “visual privacy protection” OR “personal information” OR “information privacy” OR “privacy behavio?r” OR “privacy paradox” OR “privacy calculus” OR “willingness to disclose” ) AND (video* OR camera* OR “surveillance camera*” OR “video surveillance” OR “video?monitoring” OR “monitoring technolog*” OR “camera-based technolog*” OR “video-based technolog*” OR “lifelogging camera*”) AND (“ambient assisted living” OR AAL OR “active assisted living” OR “active and healthy aging” OR “assistive needs” OR “aging in place” OR “independent living” OR “maintaining independence” OR lifelogging OR “lifelogging technolog*” OR “assistive technolog*” OR “computer vision” OR “image processing” OR gerontechnology OR “video surveillance” OR “medical monitoring” OR “health monitoring” OR “video camera*” OR “in-home monitoring” OR “smart home*” OR e?health OR telecare OR “technology acceptance” OR “perceived benefit* and barrier*” OR “perceived sensitivity” OR “personal information” OR privacy OR “information privacy” OR “privacy protection” OR “visual privacy protection” OR “privacy behaviour” OR “data security” OR anonymization OR “privacy paradox”)

*N.B. First ten pages were screened*

**SocArxiv**

***10 X 13 Results***

(elderly OR aged OR “older adult*” OR disabled OR “chronic* ill*” OR caregiver* OR “in need of care” OR “care professional*” OR “medical staff” ) AND (perception* OR attitude* OR “technology acceptance” OR privacy OR “visual privacy protection” OR “personal information” OR “information privacy” OR “privacy behavio?r” OR “privacy paradox” OR “privacy calculus” OR “willingness to disclose” ) AND (video* OR camera* OR “surveillance camera*” OR “video surveillance” OR “video?monitoring” OR “monitoring technolog*” OR “camera-based technolog*” OR “video-based technolog*” OR “lifelogging camera*”) AND (“ambient assisted living” OR AAL OR “active assisted living” OR “active and healthy aging” OR “assistive needs” OR “aging in place” OR “independent living” OR “maintaining independence” OR lifelogging OR “lifelogging technolog*” OR “assistive technolog*” OR “computer vision” OR “image processing” OR gerontechnology OR “video surveillance” OR “medical monitoring” OR “health monitoring” OR “video camera*” OR “in-home monitoring” OR “smart home*” OR e?health OR telecare OR “technology acceptance” OR “perceived benefit* and barrier*” OR “perceived sensitivity” OR “personal information” OR privacy OR “information privacy” OR “privacy protection” OR “visual privacy protection” OR “privacy behaviour” OR “data security” OR anonymization OR “privacy paradox” )

*N.B. First ten pages were screened*

**Google Scholar**

***About 77.700 results (0.08 sec)***

video* OR camera* OR “surveillance camera*” OR “video?monitoring” OR “monitoring technolog*” OR “camera-based technolog*” OR “video-based technolog*” OR “lifelogging camera*” AND privacy perceptions AND Elderly

*N.B. First ten pages were screened*
