# Supplementary material for: Acceptance and Privacy Perceptions Toward Video-based Active and Assisted Living Technologies: Scoping Review
Source: J Med Internet Res. 2023 May 1;25:e45297. doi: 10.2196/45297 (PMC10186188; doi:10.2196/45297)
Supplement: Multimedia Appendix 2 [file jmir_v25i1e45297_app2.docx]

**Appendix 2 - Inclusion and Exclusion Criteria**

|  | Include | Exclude |
| --- | --- | --- |
| *Population* | - Humans - Current and potential users of VAAL: Older adults (50+) or disabled people (of any age); Their caregivers, family members, nurses, medical staff and bystanders (of any age) |  |
| *Interest of the study* | - Video-based (RGB, depth, thermal, radar-based) monitoring technologies (including all the other technologies as long as they use video cameras) | - Video based technologies used solely for the video-conferencing / video-communications / interactive-video |
| *Context* | - Concerning privacy perception and acceptance attitudes towards camera-based monitoring technologies - Active Assisted Living / Ambient Assisted Living | - Technology used purely for medical purposes in medical hospital and not primarily AAL |
| *Physical setting of the study* | - Private home, care home, nursing home, living lab or an online survey concerning these settings | - Purely medical setting (e.g., video monitoring in emergency departments or intensive care units) |
| *Study type* | - Empirical study: qualitative or quantitative | - Theoretical study / review |
| *Year of publication* | - **∞** – 2021 August 23 |  |
| *Language* | - English, Spanish, German, French, Portuguese, Italian, Russian, Georgian. |  |
