# Supplementary material for: Acceptance and Privacy Perceptions Toward Video-based Active and Assisted Living Technologies: Scoping Review
Source: J Med Internet Res. 2023 May 1;25:e45297. doi: 10.2196/45297 (PMC10186188; doi:10.2196/45297)
Supplement: Multimedia Appendix 4 [file jmir_v25i1e45297_app4.docx]

**Acceptance and privacy perceptions toward Video-based Active and Assisted Living (VAAL) technologies – A scoping review**

**Appendix 4 -Critical Appraisal**

**Guide for the methodological quality assessment of studies that evaluate acceptability and privacy perceptions**

*This critical appraisal tool was adapted from The Methodological Quality Assessment Guide designed by* Silva et al. ***

*Questions N8 and N14 were removed from the guide as they were considered as non-applicable and the word “usability” was changed by the words “acceptability and privacy perceptions”.*

Silva, A. G., Simões, P., Santos, R., Queirós, A., Rocha, N. P., & Rodrigues, M. (2019). A Scale to Assess the Methodological Quality of Studies Assessing Usability of Electronic Health Products and Services: Delphi Study Followed by Validity and Reliability Testing. *Journal of Medical Internet Research*, *21*(11), e14829. https://doi.org/10.2196/14829

**1. Did the study use valid measurement instruments of acceptability and privacy perceptions (i.e. there is evidence that the instruments used assess acceptability and privacy perceptions)?**

This item is scored “Yes” if:

i) It is known that the instrument used was considered valid in previous studies and the study authors provide evidence of that (i.e., authors make a reference to previous studies); or

ii) It is known that the instrument used was considered valid in previous studies, but study authors do not provide evidence of that (i.e., authors make no reference to previous studies);

iii) Validity of instrument used was assessed as part of the study on acceptability and privacy perceptions;

iv) For qualitative data an effort was made to increase validity (using triangulation of methods and/or validation of the analysis and/or results by other researchers and by participants (Long & Johnson, 2000)).

This item is scored “No” if:

i) Instruments used are not considered valid or both valid and non-valid instruments are used;

ii) Authors provided insufficient information.

*Note: The most common forms of validity testing for acceptability and privacy perceptions instruments are likely to be:*

*i) construct validity (hypothesis testing) and/or ii) criterion validity. We recommended that it should be considered that there is evidence of validity in the following conditions: construct validity - the results are in accordance with pre-defined hypothesis; ii) criterion validity – correlation with a gold standard is ≥0.7 (Mokkink et al. 2018).*

| **Yes** | **No** |
| --- | --- |
| Bandini et al., 2021 [31] 🡪criterion iv |  |
| Beach et al., 2009 [32] 🡪criteria I and III |  |
|  | Berridge et al., 2019 [33] 🡪criterion II |
| Bourbonnais et al., 2019 [34] 🡪criterion iv |  |
|  | Caine et al., 2006 [35] 🡪criterion II |
|  | Caine et al., 2012 [36] 🡪criterion II |
| Demiris et al., 2004 [37] 🡪criterion iv |  |
| Gelonch et al., 2019 [38] 🡪criteria I and iv |  |
| Gövercin et al., 2016 [39] 🡪criterion I |  |
|  | Harvey et al., 2016 [40] 🡪criterion II |
|  | Lapierre et al., 2015 [41] 🡪criterion II |
| Lapierre et al., 2016 [42] 🡪criterion iv |  |
| Lapierre et al., 2018 [43] 🡪criterion iv |  |
|  | Lapierre et al., 2020 [44] 🡪criterion i |
| Londei et al., 2009 [45] 🡪criterion iv |  |
| Matthews et al., 2016 [46] 🡪criterion i |  |
|  | Mulvenna et al., 2017 [47] 🡪criterion ii |
|  | Seelye et al., 2012 [48] 🡪criterion ii |
|  | Sugihara et al., 2008 [49] 🡪criterion ii |
|  | Wilson et al., 2016 [50] 🡪criterion ii |
|  | Ziefle et al., 2011 [51] 🡪criterion ii |
|  | Ziefle et al., 2011 [52] 🡪criterion ii |

**2. Did the study use reliable measurement instruments of acceptability and privacy perceptions (i.e. there is evidence that the instruments used have similar results in repeated measures in similar circumstances)?**

This item is scored “Yes” if:

i) It is known that the instrument used was considered reliable in previous studies and the study authors provide evidence of that (i.e., authors make a reference to previous studies); or

ii) It is known that the instrument used was considered reliable in previous studies but the study authors do not provide evidence of that (i.e., authors make no reference to previous studies);

iii) Reliability of instrument used was assess as part of the study on acceptability and privacy perceptions;

iv) For qualitative data an effort was made to increase reliability (for example, using triangulation of researchers, providing the full description of the methods for data collection and analysis and accounting for personal and research method biases that may have influenced the findings).

This item is scored “No” if:

i) Instruments used are not considered reliable or both reliable and unreliable instruments are used;

ii) Authors provided insufficient information.

*Note: The most common forms of reliability testing for acceptability and privacy perceptions instruments are likely to be: i) inter-rater reliability and/or ii) test-retest reliability using either an Intraclass Correlation Coefficient (ICC) or a Weighted K. We recommended that it should be considered that there is evidence of reliability if ICC and/or weighted K ≥0.7 (Mokkink et al. 2018).*

| **Yes** | **No** |
| --- | --- |
| Bandini et al., 2021 [31] 🡪criterion iv |  |
|  | Beach et al., 2009 [32] 🡪criterion II |
|  | Berridge et al., 2019 [33] 🡪criterion II |
| Bourbonnais et al., 2019 [34] 🡪criterion iv |  |
|  | Caine et al., 2006 [35] 🡪criterion II |
|  | Caine et al., 2012 [36] 🡪criterion II |
|  |  |
| Demiris et al., 2004 [37] 🡪criterion iv |  |
| Gelonch et al., 2019 [38] 🡪criteria I and iv |  |
| Gövercin et al., 2016 [39] 🡪criterion I |  |
|  | Harvey et al., 2016 [40] 🡪criterion II |
|  | Lapierre et al., 2015 [41] 🡪criterion II |
| Lapierre et al., 2016 [42] 🡪criterion iv |  |
| Lapierre et al., 2018 [43] 🡪criterion iv |  |
|  | Lapierre et al., 2020 [44] 🡪criterion i |
|  | Londei et al., 2009 [45] 🡪criterion ii |
| Matthews et al., 2016 [46] 🡪criterion i |  |
|  | Mulvenna et al., 2017 [47] 🡪criterion ii |
|  | Seelye et al., 2012 [48] 🡪criterion ii |
|  | Sugihara et al., 2008 [49] 🡪criterion ii |
|  | Wilson et al., 2016 [50] 🡪criterion ii |
|  | Ziefle et al., 2011 [51] 🡪criterion ii |
|  | Ziefle et al., 2011 [52] 🡪criterion ii |

**3. Was there coherence between the procedures used to assess acceptability and privacy perceptions and (e.g. instruments, context, …) and study aims?**

This item is scored “Yes” if:

i) The procedures to assess the acceptability and privacy perceptions and were chosen in accordance to the objectives of the study (for example, if the aim is to gather the subjective perception of participants, a more qualitative approach may be appropriate; if the aim is to collect data from a larger number of participants on a fully functional product a more quantitative assessment may be appropriate).

This item is scored “No” if:

1. Procedures to assess acceptability and privacy perceptions were not coherent with study aims; or
2. Authors provided insufficient information.

| **Yes** | **No** |
| --- | --- |
| Bandini et al., 2021 [31] 🡪criterion I |  |
| Beach et al., 2009 [32] 🡪criterion I |  |
| Berridge et al., 2019 [33] 🡪criterion I |  |
| Bourbonnais et al., 2019 [34] 🡪criterion I |  |
| Caine et al., 2006 [35] 🡪criterion I |  |
| Caine et al., 2012 [36] 🡪criterion I |  |
| Demiris et al., 2004 [37] 🡪criterion I |  |
| Gelonch et al., 2019 [38] 🡪criterion I |  |
| Gövercin et al., 2016 [39] 🡪criterion I |  |
| Harvey et al., 2016 [40] 🡪criterion I |  |
| Lapierre et al., 2015 [41] 🡪criterion I |  |
| Lapierre et al., 2016 [42] 🡪criterion I |  |
| Lapierre et al., 2018 [43] 🡪criterion I |  |
| Lapierre et al., 2020 [44] 🡪criterion I |  |
| Londei et al., 2009 [45] 🡪criterion i |  |
| Matthews et al., 2016 [46] 🡪criterion i |  |
| Mulvenna et al., 2017 [47] 🡪criterion I |  |
| Seelye et al., 2012 [48] 🡪criterion i |  |
| Sugihara et al., 2008 [49] 🡪criterion i |  |
| Wilson et al., 2016 [50] 🡪criterion i |  |
| Ziefle et al., 2011 [51] 🡪criterion i |  |
| Ziefle et al., 2011 [52] 🡪criterion i |  |

**4. Did the study use procedures of assessment for acceptability and privacy perceptions that were adequate to the development stage of the product/service?**

This item is scored “Yes” if:

i) The procedures to assess acceptability and privacy perceptions were adequate to the stage of development of the product (for example, in the beginning of the product/service development, it is expected that acceptability and privacy perceptions assessments are performed laboratory environment and using experts; for a mature service/product it is expected that acceptability and privacy perceptions is assessed in real context with potential end users).

This item is scored “No” if:

1. Procedures to assess acceptability and privacy perceptions were not adequate to the stage of development of the product; or
2. Authors provided insufficient information.

| **Yes** | **No** |
| --- | --- |
| Bandini et al., 2021 [31] 🡪criterion I |  |
| Beach et al., 2009 [32] 🡪criterion I |  |
|  | Berridge et al., 2019 [33] 🡪criterion II |
| Bourbonnais et al., 2019 [34] 🡪criterion I |  |
| Caine et al., 2006 [35] 🡪criterion I |  |
| Caine et al., 2012 [36] 🡪criterion I |  |
| Demiris et al., 2004 [37] 🡪criterion I |  |
| Gelonch et al., 2019 [38] 🡪criterion I |  |
| Gövercin et al., 2016 [39] 🡪criterion I |  |
| Harvey et al., 2016 [40] 🡪criterion I |  |
| Lapierre et al., 2015 [41] 🡪criterion I |  |
| Lapierre et al., 2016 [42] 🡪criterion I |  |
| Lapierre et al., 2018 [43] 🡪criterion I |  |
| Lapierre et al., 2020 [44] 🡪criterion I |  |
| Londei et al., 2009 [45] 🡪criterion i |  |
| Matthews et al., 2016 [46] 🡪criterion i |  |
| Mulvenna et al., 2017 [47] 🡪criterion I |  |
| Seelye et al., 2012 [48] 🡪criterion i |  |
| Sugihara et al., 2008 [49] 🡪criterion i |  |
| Wilson et al., 2016 [50] 🡪criterion i |  |
| Ziefle et al., 2011 [51] 🡪criterion i |  |
| Ziefle et al., 2011 [52] 🡪criterion i |  |

**5. Did the study use procedures of assessment for acceptability and privacy perceptions adequate to study participants’ characteristics?**

This item is scored “Yes” if:

i) The procedures of assessment for acceptability and privacy perceptions was adequate to study participants’ characteristics, particularly: age, cognitive function, educational level, clinical condition, technological literacy.

This item is scored “No” if:

i) Procedures to assess acceptability and privacy perceptions were not adequate to study participants’ characteristics; or

ii) Authors provided insufficient information.

| **Yes** | **No** |
| --- | --- |
| Bandini et al., 2021 [31] 🡪criterion I |  |
| Beach et al., 2009 [32] 🡪criterion I |  |
| Berridge et al., 2019 [33] 🡪criterion I |  |
| Bourbonnais et al., 2019 [34] 🡪criterion I |  |
| Caine et al., 2006 [35] 🡪criterion I |  |
| Caine et al., 2012 [36] 🡪criterion I |  |
| Demiris et al., 2004 [37] 🡪criterion I |  |
| Gelonch et al., 2019 [38] 🡪criterion I |  |
| Gövercin et al., 2016 [39] 🡪criterion I |  |
| Harvey et al., 2016 [40] 🡪criterion I |  |
| Lapierre et al., 2015 [41] 🡪criterion I |  |
| Lapierre et al., 2016 [42] 🡪criterion I |  |
| Lapierre et al., 2018 [43] 🡪criterion I |  |
| Lapierre et al., 2020 [44] 🡪criterion I |  |
| Londei et al., 2009 [45] 🡪criterion i |  |
| Matthews et al., 2016 [46] 🡪criterion i |  |
| Mulvenna et al., 2017 [47] 🡪criterion I |  |
| Seelye et al., 2012 [48] 🡪criterion i |  |
|  | Sugihara et al., 2008 [49] 🡪criterion ii |
| Wilson et al., 2016 [50] 🡪criterion i |  |
| Ziefle et al., 2011 [51] 🡪criterion i |  |
| Ziefle et al., 2011 [52] 🡪criterion i |  |
|  |  |

**6. Did the study employ triangulation of methods for the assessment of acceptability and privacy perceptions?**

This item is scored “Yes” if:

i) The study used a combination of at least two methods, one qualitative (for example, interviews) and the other quantitative (for example, questionnaires) to assess acceptability and privacy perceptions (across method triangulation); or

ii) The study used a combination of at least two methods, both qualitative or quantitative, to assess the acceptability and privacy perceptions (within-method triangulation).

This item is scored “No” if:

1. Only one method was used to assess acceptability and privacy perceptions; or

ii) Authors provided insufficient information.

| **Yes** | **No** |
| --- | --- |
| Bandini et al., 2021 [31] 🡪criterion ii |  |
|  | Beach et al., 2009 [32] 🡪criterion I |
|  | Berridge et al., 2019 [33] 🡪criterion II |
|  | Bourbonnais et al., 2019 [34] 🡪criterion I |
|  | Caine et al., 2006 [35] 🡪criterion II |
|  | Caine et al., 2012 [36] 🡪criterion I |
|  | Demiris et al., 2004 [37] 🡪criterion I |
| Gelonch et al., 2019 [38] 🡪criterion i |  |
| Gövercin et al., 2016 [39] 🡪criterion i |  |
| Harvey et al., 2016 [40] 🡪criterion ii |  |
| Lapierre et al., 2015 [41] 🡪criterion i |  |
|  | Lapierre et al., 2016 [42] 🡪criterion I |
|  | Lapierre et al., 2018 [43] 🡪criterion i |
| Lapierre et al., 2020 [44] 🡪criterion i |  |
| Londei et al., 2009 [45] 🡪criterion i |  |
| Matthews et al., 2016 [46] 🡪criterion i |  |
|  | Mulvenna et al., 2017 [47] 🡪criterion i |
|  | Seelye et al., 2012 [48] 🡪criterion i |
|  | Sugihara et al., 2008 [49] 🡪criterion i |
|  | Wilson et al., 2016 [50] 🡪criterion i |
| Ziefle et al., 2011 [51] 🡪criterion i |  |
|  | Ziefle et al., 2011 [52] 🡪criterion i |

**7. Was the type of analysis adequate to the study’s aims and variables measurement scale?**

This item is scored “Yes” if:

i) It is clear how the data was assessed, and the type of analyses was adequate (for example, content analysis for qualitative data and the appropriate statistical tests for quantitative data).

This item is scored “No” if:

i) The analysis was not appropriate (either a quantitative or a qualitative approach) or the statistical test used (quantitative data) was not the most appropriate.

ii) Authors provided insufficient information on the type of analysis performed (for example, they reported on using thematic analysis for qualitative data, but did not describe how the analysis was performed).

| **Yes** | **No** |
| --- | --- |
| Bandini et al., 2021 [31] 🡪criterion I |  |
| Beach et al., 2009 [32] 🡪criterion I |  |
|  | Berridge et al., 2019 [33] 🡪criterion II |
| Bourbonnais et al., 2019 [34] 🡪criterion I |  |
|  | Caine et al., 2006 [35] 🡪criterion II |
| Caine et al., 2012 [36] 🡪criterion I |  |
| Demiris et al., 2004 [37] 🡪criterion I |  |
| Gelonch et al., 2019 [38] 🡪criterion I |  |
| Gövercin et al., 2016 [39] 🡪criterion I |  |
| Harvey et al., 2016 [40] 🡪criterion I |  |
| Lapierre et al., 2015 [41] 🡪criterion I |  |
| Lapierre et al., 2016 [42] 🡪criterion I |  |
| Lapierre et al., 2018 [43] 🡪criterion I |  |
| Lapierre et al., 2020 [44] 🡪criterion I |  |
| Londei et al., 2009 [45] 🡪criterion i |  |
| Matthews et al., 2016 [46] 🡪criterion i |  |
|  | Mulvenna et al., 2017 [47] 🡪criterion ii |
|  | Seelye et al., 2012 [48] 🡪criterion ii |
|  | Sugihara et al., 2008 [49] 🡪criterion ii |
| Wilson et al., 2016 [50] 🡪criterion i |  |
| Ziefle et al., 2011 [51] 🡪criterion i |  |
| Ziefle et al., 2011 [52] 🡪criterion i |  |

**8. Were participants who assessed the product/service acceptability and privacy perceptions representative of the potential user’s population?**

This item is scored “Yes” if:

i) Participants were representative of the population of experts and/or potential users. A minimal set of data should be given for experts (age, sex, area of expertise/professional occupation, years of practice, where they were recruited from) and users (age, sex, educational level, asymptomatic/clinical condition, where they were recruited from).

This item is scored “No” if:

i) Participants were not representative of the population of potential users; or

ii) Authors provided insufficient information.

| **Yes** | **No** |
| --- | --- |
| Bandini et al., 2021 [31] 🡪criterion I |  |
| Beach et al., 2009 [32] 🡪criterion I |  |
| Berridge et al., 2019 [33] 🡪criterion I |  |
| Bourbonnais et al., 2019 [34] 🡪criterion I |  |
| Caine et al., 2006 [35] 🡪criterion I |  |
| K. Caine et al., 2012 [36] 🡪criterion I |  |
| Demiris et al., 2004 [37] 🡪criterion I |  |
| Gelonch et al., 2019 [38] 🡪criterion I |  |
| Gövercin et al., 2016 [39] 🡪criterion I |  |
| Harvey et al., 2016 [40] 🡪criterion I |  |
| Lapierre et al., 2015 [41] 🡪criterion I |  |
| Lapierre et al., 2016 [42] 🡪criterion I |  |
| Lapierre et al., 2018 [43] 🡪criterion I |  |
| Lapierre et al., 2020 [44] 🡪criterion I |  |
| Londei et al., 2009 [45] 🡪criterion i |  |
| Matthews et al., 2016 [46] 🡪criterion i |  |
| Mulvenna et al., 2017 [47] 🡪criterion i |  |
| Seelye et al., 2012 [48] 🡪criterion i |  |
| Sugihara et al., 2008 [49] 🡪criterion i |  |
| Wilson et al., 2016 [50] 🡪criterion i |  |
|  | Ziefle et al., 2011 [51] 🡪criterion i |
|  | Ziefle et al., 2011 [52] 🡪criterion i |

**9. Was the investigator that conducted the qualitative assessment of acceptability and privacy perceptions assessments adequately trained?**

This item is scored “Yes” if:

i) The study refers that the investigator conducting the qualitative assessment of acceptability and privacy perceptions had previous experience of using qualitative assessment methods (for example, had already conducted at least one acceptability and privacy perceptions assessment using the same method) or was trained to do it.

This item is scored “No” if:

1. The study refers that the investigator conducting the acceptability and privacy perceptions assessment had no/insufficient previous experience;
2. Authors provided insufficient information.

| **Yes** | **No** |
| --- | --- |
|  | Bandini et al., 2021 [31] 🡪criterion II |
|  | Beach et al., 2009 [32] 🡪criterion II |
|  | Berridge et al., 2019 [33] 🡪criterion II |
|  | Bourbonnais et al., 2019 [34] 🡪criterion II |
|  | Caine et al., 2006 [35] 🡪criterion II |
|  | K. Caine et al., 2012 [36] 🡪criterion II |
|  | Demiris et al., 2004 [37] 🡪criterion II |
|  | Gelonch et al., 2019 [38] 🡪criterion II |
|  | Gövercin et al., 2016 [39] 🡪criterion II |
|  | Harvey et al., 2016 [40] 🡪criterion II |
|  | Lapierre et al., 2015 [41] 🡪criterion II |
|  | Lapierre et al., 2016 [42] 🡪criterion II |
|  | Lapierre et al., 2018 [43] 🡪criterion ii |
|  | Lapierre et al., 2020 [44] 🡪criterion ii |
|  | Londei et al., 2009 [45] 🡪criterion ii |
|  | Matthews et al., 2016 [46] 🡪criterion ii |
|  | Mulvenna et al., 2017 [47] 🡪criterion ii |
|  | Seelye et al., 2012 [48] 🡪criterion ii |
|  | Sugihara et al., 2008 [49] 🡪criterion ii |
|  | Wilson et al., 2016 [50] 🡪criterion ii |
|  | Ziefle et al., 2011 [51] 🡪criterion ii |
|  | Ziefle et al., 2011 [52] 🡪criterion ii |

**10. Was the investigator that conducted acceptability and privacy perceptions assessments external to the process of product/service development?**

This item is scored “Yes” if:

i) The study refers that the investigator conducting the acceptability and privacy perceptions assessment was not involved in the development of the product.

This item is scored “No” if:

i) The study refers that the investigator conducting acceptability and privacy perceptions assessments was involved in the development of the product; or

ii) Authors provided insufficient information.

| **Yes** | **No** |
| --- | --- |
|  | Bandini et al., 2021 [31] 🡪criterion II |
|  | Beach et al., 2009 [32] 🡪criterion II |
|  | Berridge et al., 2019 [33] 🡪criterion II |
|  | Bourbonnais et al., 2019 [34] 🡪criterion II |
|  | Caine et al., 2006 [35] 🡪criterion II |
|  | K. Caine et al., 2012 [36] 🡪criterion II |
|  | Demiris et al., 2004 [37] 🡪criterion II |
|  | Gelonch et al., 2019 [38] 🡪criterion II |
|  | Gövercin et al., 2016 [39] 🡪criterion II |
|  | Harvey et al., 2016 [40] 🡪criterion II |
|  | Lapierre et al., 2015 [41] 🡪criterion II |
|  | Lapierre et al., 2016 [42] 🡪criterion II |
|  | Lapierre et al., 2018 [43] 🡪criterion ii |
|  | Lapierre et al., 2020 [44] 🡪criterion ii |
|  | Londei et al., 2009 [45] 🡪criterion ii |
|  | Matthews et al., 2016 [46] 🡪criterion ii |
|  | Mulvenna et al., 2017 [47] 🡪criterion ii |
|  | Seelye et al., 2012 [48] 🡪criterion ii |
|  | Sugihara et al., 2008 [49] 🡪criterion ii |
|  | Wilson et al., 2016 [50] 🡪criterion ii |
|  | Ziefle et al., 2011 [51] 🡪criterion ii |
|  | Ziefle et al., 2011 [52] 🡪criterion ii |

**11. Was the acceptability and privacy perceptions assessment conducted in the real context or close to the real context where product/service is going to be used?***

This item is scored “Yes” if:

i) The study refers that the acceptability and privacy perceptions assessments were conducted in the context (or at least close to) in which the product is going to be used.

This item is scored “No” if:

1. The study refers that acceptability and privacy perceptions assessment s were conducted in laboratory or in a context different from the context where the product/service is going to be used; or

ii) Authors provided insufficient information.

| **Yes** | **No** |
| --- | --- |
| Bandini et al., 2021 [31] 🡪criterion I |  |
|  | Beach et al., 2009 [32] 🡪criterion I |
| Berridge et al., 2019 [33] 🡪criterion I |  |
| Bourbonnais et al., 2019 [34] 🡪criterion I |  |
|  | Caine et al., 2006 [35] 🡪criterion II |
|  | Caine et al., 2012 [36] 🡪criterion I |
| Demiris et al., 2004 [37] 🡪criterion I |  |
| Gelonch et al., 2019 [38] 🡪criterion I |  |
| Gövercin et al., 2016 [39] 🡪criterion I |  |
| Harvey et al., 2016 [40] 🡪criterion I |  |
| Lapierre et al., 2015 [41] 🡪criterion I |  |
|  | Lapierre et al., 2016 [42] 🡪criterion I |
| Lapierre et al., 2018 [43] 🡪criterion i |  |
| Lapierre et al., 2020 [44] 🡪criterion i |  |
| Londei et al., 2009 [45] 🡪criterion i |  |
| Matthews et al., 2016 [46] 🡪criterion i |  |
|  | Mulvenna et al., 2017 [47] 🡪criterion i |
| Seelye et al., 2012 [48] 🡪criterion i |  |
| Sugihara et al., 2008 [49] 🡪criterion i |  |
| Wilson et al., 2016 [50] 🡪criterion i |  |
|  | Ziefle et al., 2011 [51] 🡪criterion i |
|  | Ziefle et al., 2011 [52] 🡪criterion i |

**12. Was the number of participants used to assess acceptability and privacy perceptions adequate (whether potential users or experts)?**

This item is scored “Yes” if:

i) The study performed an a priori sample size calculation for quantitative assessments (for example, estimative of the sample size); or authors provide evidence that they reached the saturation point for qualitative studies;

ii) The study justifies sample size based on recommendations (for example, for formative evaluation a sample size of 5 to 10 participants are considered to be sufficient while for summative evaluations is necessary at least a sample size of 30 participants (Lewis, 2014)).

This item is scored “No” if:

i) The sample size used in the study was not justified or was considered small.

| **Yes** | **No** |
| --- | --- |
|  | Bandini et al., 2021 [31] 🡪criterion I |
| Beach et al., 2009 [32] 🡪criterion II |  |
|  | Berridge et al., 2019 [33] 🡪criterion I |
|  | Bourbonnais et al., 2019 [34] 🡪 criterion I |
|  | Caine et al., 2006 [35] 🡪 criterion I |
|  | Caine et al., 2012 [36] 🡪 criterion I |
|  | Demiris et al., 2004 [37] 🡪not justified |
|  | Gelonch et al., 2019 [38] 🡪criterion I |
|  | Gövercin et al., 2016 [39] 🡪 criterion I |
|  | Harvey et al., 2016 [40] 🡪 criterion I |
|  | Lapierre et al., 2015 [41] 🡪 criterion I |
|  | Lapierre et al., 2016 [42] 🡪 criterion I |
|  | Lapierre et al., 2018 [43] 🡪criterion i |
|  | Lapierre et al., 2020 [44] 🡪criterion i |
|  | Londei et al., 2009 [45] 🡪criterion i |
|  | Matthews et al., 2016 [46] 🡪criterion i |
|  | Mulvenna et al., 2017 [47] 🡪criterion i |
|  | Seelye et al., 2012 [48] 🡪criterion i |
|  | Sugihara et al., 2008 [49] 🡪criterion i |
|  | Wilson et al., 2016 [50] 🡪criterion i |
|  | Ziefle et al., 2011 [51] 🡪criterion i |
|  | Ziefle et al., 2011 [52] 🡪criterion i |

**13. Was the acceptability and privacy perceptions assessment based on continuous and prolonged use of the product/service over time?**

This item is scored “Yes” if:

i) The product/service was use for several hours or days in the real context.

This item is scored “No” if:

i) The product/service was used for a very limited period of time in the presence of the investigator, usually with a pre-defined task to complete;

ii) Authors provided insufficient information.

| **Yes** | **No** |
| --- | --- |
| Bandini et al., 2021 [31] 🡪criterion I |  |
|  | Beach et al., 2009 [32] 🡪criterion I |
|  | Berridge et al., 2019 [33] 🡪criterion I |
|  | Bourbonnais et al., 2019 [34] 🡪criterion I |
|  | Caine et al., 2006 [35] 🡪criterion I |
| Caine et al., 2012 [36] 🡪criterion I |  |
|  | Demiris et al., 2004 [37] 🡪criterion i |
| Gelonch et al., 2019 [38] 🡪criterion I |  |
| Gövercin et al., 2016 [39] 🡪criterion I |  |
| Harvey et al., 2016 [40] 🡪criterion I |  |
|  | Lapierre et al., 2015 [41] 🡪criterion I |
|  | Lapierre et al., 2016 [42] 🡪criterion I |
| Lapierre et al., 2018 [43] 🡪criterion i |  |
| Lapierre et al., 2020 [44] 🡪criterion i |  |
|  | Londei et al., 2009 [45] 🡪criterion i |
| Matthews et al., 2016 [46] 🡪criterion i |  |
|  | Mulvenna et al., 2017 [47] 🡪criterion i |
| Seelye et al., 2012 [48] 🡪criterion i |  |
| Sugihara et al., 2008 [49] 🡪criterion i |  |
| Wilson et al., 2016 [50] 🡪criterion i |  |
|  | Ziefle et al., 2011 [51] 🡪criterion i |
|  | Ziefle et al., 2011 [52] 🡪criterion i |

**Summary results:**

| **Study** | **Quality Assessment Score (out of 13)** |
| --- | --- |
| Bandini et al., 2021 | 10 |
| Beach et al., 2009 | 7 |
| Berridge et al., 2019 | 4 |
| Bourbonnais et al., 2019 | 8 |
| Caine et al., 2006 | 4 |
| K. Caine et al., 2012 | 6 |
| Demiris et al., 2004 | 8 |
| Gelonch et al., 2019 | 10 |
| Gövercin et al., 2016 | 10 |
| Harvey et al., 2016 | 8 |
| Lapierre et al., 2015 | 7 |
| Lapierre et al., 2016 | 7 |
| Lapierre et al., 2018 | 9 |
| Lapierre et al., 2020 | 8 |
| Londei et al., 2009 | 8 |
| Matthews et al., 2016 | 10 |
| Mulvenna et al., 2017 | 4 |
| Seelye et al., 2012 | 6 |
| Sugihara et al., 2008 | 5 |
| Wilson et al., 2016 | 8 |
| Ziefle et al., 2011 | 4 |
| Ziefle et al., 2011 | 4 |

| **Score** | **N of studies** | **Percentage** |
| --- | --- | --- |
| 10 | 4 | 18.2% |
| 9 | 1 | 4.5% |
| 8 | 6 | 27.3% |
| 7 | 3 | 13.6% |
| 6 | 2 | 9.1% |
| 5 | 1 | 4.5% |
| 4 | 5 | 22.7% |
